# Anti-Obesity Effects of Marine Macroalgae Extract *Caulerpa lentillifera* in a *Caenorhabditis elegans* Model

**DOI:** 10.3390/md21110577

**Published:** 2023-11-03

**Authors:** Kawita Chumphoochai, Preeyanuch Manohong, Nakorn Niamnont, Montakan Tamtin, Prasert Sobhon, Krai Meemon

**Affiliations:** 1 Department of Anatomy, Faculty of Science, Mahidol University, Rama VI Road, Bangkok 10400, Thailand; kawita.ch@gmail.com (K.C.); prasertsobhon@gmail.com (P.S.); 2 Department of Chemistry, Faculty of Science, King Mongkut’s University of Technology Thonburi, Bang Mod, Bangkok 10140, Thailand; preeyanuch_m01@hotmail.co.th (P.M.); nakorn.nia@kmutt.ac.th (N.N.); 3 Kung Krabaen Bay Royal Development of Fisheries, Khlong Khut Sub-District, Tha Mai, Chantaburi 22000, Thailand; mtamtin@hotmail.com

**Keywords:** obesity, fat accumulation, macroalgae, green seaweed, *Caulerpa lentillifera*, sea grape, *Caenorhabditis elegans*

## Abstract

Obesity is a multifactorial disease characterized by an excessive accumulation of fat, which in turn poses a significant risk to health. Bioactive compounds obtained from macroalgae have demonstrated their efficacy in combating obesity in various animal models. The green macroalgae *Caulerpa lentillifera* (CL) contains numerous active constituents. Hence, in the present study, we aimed to elucidate the beneficial anti-obesity effects of extracts derived from *C. lentillifera* using a *Caenorhabditis elegans* obesity model. The ethanol (CLET) and ethyl acetate (CLEA) extracts caused a significant decrease in fat consumption, reaching up to approximately 50–60%. Triglyceride levels in 50 mM glucose-fed worms were significantly reduced by approximately 200%. The GFP-labeled *dhs-3*, a marker for lipid droplets, exhibited a significant reduction in its level to approximately 30%. Furthermore, the level of intracellular ROS displayed a significant decrease of 18.26 to 23.91% in high-glucose-fed worms treated with CL extracts, while their lifespan remained unchanged. Additionally, the mRNA expression of genes associated with lipogenesis, such as *sbp-1*, showed a significant down-regulation following treatment with CL extracts. This finding was supported by a significant decrease (at 16.22–18.29%) in GFP-labeled *sbp-1* gene expression. These results suggest that *C. lentillifera* extracts may facilitate a reduction in total fat accumulation induced by glucose through *sbp-1* pathways. In summary, this study highlights the anti-obesity potential of compounds derived from *C. lentillifera* extracts in a *C. elegans* model of obesity, mediated by the suppression of lipogenesis pathways.

## 1. Introduction

Obesity is emerging as a global pandemic in the 21st century due to the rising prevalence of overweight individuals and excessive weight gain. The global prevalence has nearly doubled since 1980 [1]. It has been reported that more than 1.9 billion and 650 million people worldwide were overweight and obese in 2016, respectively [2]. The World Obesity Federation has forecasted that by 2035, the global population of overweight and obese individuals could surpass 4 billion, marking a significant rise from the 2.6 billion recorded in 2020. This represents an alarming increase, with the percentage of the world’s population affected by overweight and obesity, previously at 38% in 2020, projected to surge to 50% by 2035 [3]. The World Health Organization (WHO) defines obesity as abnormal or excessive fat accumulation that can contribute to a high risk of metabolic diseases [4]. Body mass index (BMI) is a simple tool to classify a person into one of the underweight, normal weight, overweight, and obese categories. A BMI of 25 to 29.9 is categorized as overweight, and a BMI of 30 to 35 or greater is categorized as obese [5]. Increasing BMI is an important factor enhancing the risk of various chronic diseases, such as type 2 diabetes, hypertension, cardiovascular disease, osteoarthritis, and some types of cancers [2,6]. These medical problems affect quality of life and increase the risk of death in obese patients [7,8]. Therefore, the majority of countries around the world have launched campaigns to decrease and prevent obesity, such as education, exercise, and diet control plans. However, several obese patients fail to achieve this long-term goal due to many factors that affect their bodies’ responses to weight loss. Pharmacological options have been recommended for patients with BMIs ≥ 30 for whom lifestyle changes are not effective [9]. There are various drugs that have been approved by the U.S. FDA (Food and Drug Administration) for short-term use, but only five have been approved for long-term treatment due to their side effects [10,11,12]. Hence, further exploration of potential anti-obesity drugs and compounds are urgently needed.

Marine macroalgae (also known as seaweeds) contain various nutrients and biologically active components depending on the species, origin of growth, time of harvest, and environmental conditions [13,14]. There are more than 600 species of edible seaweed with a great variety of macronutrients; micronutrients; and bioactive compounds with valuable pharmaceutical potential, such as polyphenols, polysaccharides, and amino acids [12,13,14,15]. These compounds exhibit many biological activities, such as anti-oxidation, anti-aging effects, anti-tumor activity, photoprotective action, and decreasing lipid accumulation [16,17].

*Caulerpa lentillifera* (*C. lentillifera*) is a type of ulvophyte green macroalgae with a grape-like appearance belonging to the *Caulerpa* genus. It originated in the tropical locations of the Indian and Pacific oceans. It can also be found in various countries due to the alteration of global temperatures, such as Korea, Japan, Taiwan, Oceania, and Southeast Asia [15,16,17,18]. It contains many bioactive compounds, including phenolic compounds, polysaccharides, and siphonaxanthin [18]. *C. lentillifera* has gained popularity among consumers in some Asian countries due to its good taste; nutritional value; and significant therapeutic potential thanks to its anti-inflammatory, anti-oxidant, anti-cancer, and anti-obesity activities [18,19,20]. Supplementation with *C. lentillifera*-derived extracts (CL) for 16 weeks showed anti-obesity activity in rats fed with a high-cholesterol diet. These extracts significantly increased HDL-C levels and reduced LDL-C and triglyceride levels, along with decreased body weight, systolic blood pressure, and total cholesterol [21,22]. However, the mechanism of action of bioactive compounds derived from *C. lentillifera* for anti-obesity activity have not yet been studied.

Therefore, this study aimed to investigate the effects of *C. lentillifera* extracts on anti-obesity activity, as well as the mechanisms of their actions, using an in vivo *Caenorhabditis elegans* (*C. elegans*) model. *C. elegans* is a free-living soil nematode that is widely employed as a model in biomedical research, including obesity studies. Its conserved set of genes for fat storage and metabolism are similar to those found in humans. In addition, *C. elegans* contains types of fatty acids similar to those found in mammals [23]. Therefore, *C. elegans* is an appropriate animal model for an obesity versus anti-obesity investigation such as that described in the present study. The positive anti-obesity properties of the extracts from this study hold the potential to provide valuable insights into the application of *C. lentillifera* extracts for combating obesity.

## 2. Results

### 2.1. Toxicity of the Extracts from C. lentillifera

Doses of CL extracts were evaluated before the experiments using the dose–response assessment, which describes the relationship between the intensity of exposure and the probability of a detrimental health response [24]. We found that the survival rates of the worms exposed to CL extracts at 100, 250, 500, 750, and 1000 µg/mL were 100%, indicating that no fractions of CL extracts are toxic to *C. elegans* (Figure S1). Thus, the dose–response curve was generated, and the lethal dose at 50% (LD50) could not be calculated. Furthermore, the lifespan analysis (Figure S2) verified the safety of the doses at 100, 500, and 1000 µg/mL. Notably, when subjected to CL treatment alone, all worms displayed normal lifespans, without any discernible reduction. Consequently, the doses of 100, 500, and 1000 µg/mL were chosen in order to investigate the effects of CL extracts on fat accumulation in *C. elegans*. 

### 2.2. CL Extracts Decrease Total Fat Accumulation in High Glucose-Fed C. elegans

#### 2.2.1. Fat Deposition in High Glucose-Fed *C. elegans*

**Figure 1:**
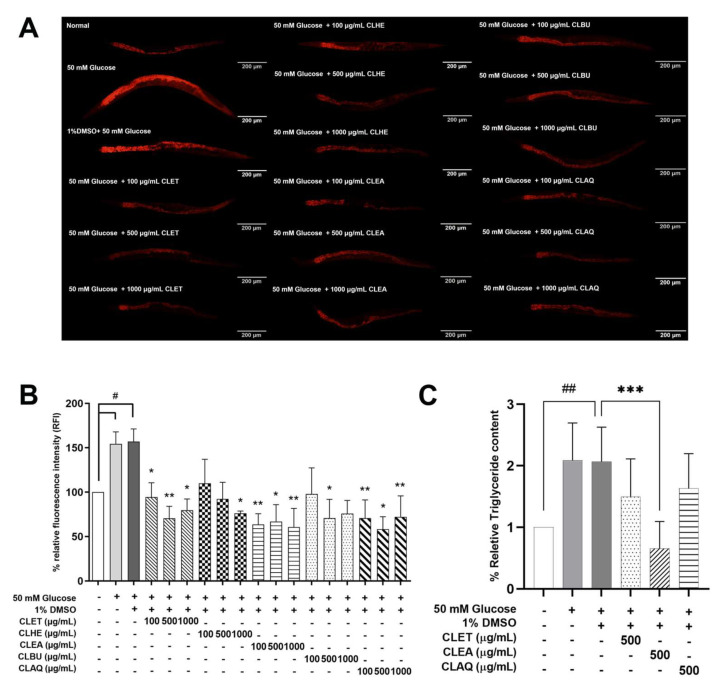
Effects of *C. lentillifera* extracts in terms of reducing fat accumulation and triglyceride contents mainly stored in the intestine of a *C. elegans* model of obesity. (**A**) Nile red fluorescence intensity of 50 mM glucose-fed worms treated with CL extracts. (**B**) Graphical representation of the relative Nile red fluorescence intensity of 50 mM glucose-fed *C. elegans* treated with CL extracts. (**C**) Graphical representation of relative triglyceride contents in 50 mM glucose-fed worms treated with CL extracts. Asterisks (*), (**), and (***) indicate significant differences at *p*-values < 0.05, 0.01, and 0.001, respectively. Sharps (#) and (##) indicate significant differences at *p*-values < 0.05 and 0.01 between the glucose-fed and normal groups.

For the use of 50 mM glucose, we followed the methodology of the previous study [25] to induce fat accumulation in a *C. elegans* model of obesity. Figure 1A,B shows that the mean fluorescence intensities of Nile red in the worms fed with 50 mM glucose, with or without DMSO (1% final concentration), were significantly increased to 54.29% and 57.08%, respectively, compared with the normal worms, suggesting that 50 mM glucose-fed worms can be used in an obesity model. Moreover, there was no difference between these two groups, indicating that 1% DMSO had no effect on fat accumulation in *C. elegans*. Thus, the worms treated with 50 mM glucose and 1% DMSO were used as the control groups. After CL treatment, the high-glucose-fed worms exhibited a decrease in total fat accumulation, especially those treated with the CLET, CLEA, and CLAQ extracts (Figure 1A,B). The mean fluorescence intensities of Nile red staining in glucose-fed worms treated with ethanolic fractions of CL (CLET) were significantly decreased to 39.9%, 55.08%, and 20.47% for doses of 100, 500, and 1000 µg/mL, respectively. For the ethyl acetate fraction of the CL extracts (CLEA), the mean fluorescence intensities were significantly decreased to 59.41%, 57.39%, and 61.42% for the doses of 100, 500, and 1000 µg/mL, respectively. Similarly, the mean fluorescence intensities of the aqueous fraction of CL extracts (CLAQ) were significantly decreased to 54.93%, 62.87%, and 54.02% for the doses of 100, 500, and 1000 µg/mL, respectively. The results demonstrate that the CLET, CLEA, and CLAQ extracts were effective in ameliorating fat accumulation in high-glucose-fed *C. elegans*, and thus, they were used in the subsequent experiments.

#### 2.2.2. Triglyceride Levels in High-Glucose-Fed *C. elegans*

Triglycerides (TG) are a type of dietary fat which is synthesized from fatty acids within cells and then stored in the body [26]. To examine whether CL extracts decrease TG levels in high-glucose-fed *C. elegans*, the triglyceride levels were measured using a triglyceride quantification kit (Biovision, California, USA). According to the Nile red assay, 500 µg/mL extracts demonstrated greater effectiveness than other doses, especially CLET, CLEA, and CLAQ. Consequently, 500 µg/mL samples of these extracts were selected for subsequent experiments. High-glucose-fed worms were treated with CLET, CLEA, and CLAQ at 500 µg/mL, then incubated at 20 °C for 96 h. The high-glucose-fed worms, with or without DMSO, showed higher levels of triglyceride content than the normal group. After CL treatment, the levels of triglycerides were significantly decreased to 215.08% for 500 µg/mL CLEA compared to the 1% DMSO control group (Figure 1C). However, it was observed that CLAQ was less effective in reducing the triglyceride content. Thereby, CLET and CLEA were chosen for further investigation into the anti-obesity activity of the CL extracts.

#### 2.2.3. GFP-Labeled *dhs-3* Expression Levels in High Glucose-Fed *C. elegans*

**Figure 2:**
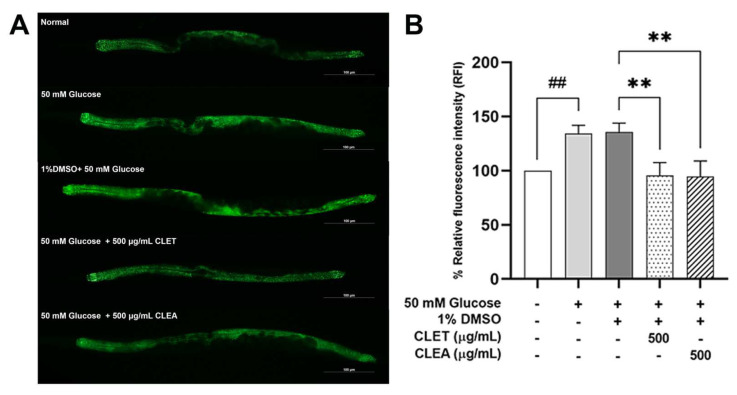
*C. lentillifera* extracts decreasing the expression level of the *dhs-3* gene in 50 mM glucose-fed *C. elegans*. (**A**) Expression pattern of GFP-labeled *dhs-3* gene. (**B**) The relative fluorescence intensity, in percentage, of GFP-labeled *dhs-3* expression. The data represent the mean ± SEM (n = 40 per group, number of animals), measured using ImageJ software. Asterisks (**) indicate significant differences at a *p*-value < 0.01. A double sharp (##) denotes a significant difference at a *p*-value < 0.01 between the glucose-fed and normal groups.

Lipid droplets serve as fat storage organelles in *C. elegans,* and are located in the intestine [27,28]. To determine the expression levels of *dhs-3,* which is a marker of lipid droplet in worms, LIU-1 transgenic strain (*dhs-3*p::*dhs-3*::GFP + *unc-76*(+)) was used. We found a significant decrease in the mean fluorescence intensity in both of the 500 µg/mL CLET and CLEA treatments at 29.57 and 30.29%, respectively (Figure 2). This indicates that CL extracts can reduce TG levels, leading to a decrease in the lipid droplet numbers. 

### 2.3. Gas Chromatography–Mass Spectrometry (GC-MS) of CLET and CLEA

To identify compounds contained in CLET and CLEA, gas chromatography–mass spectrometry (GC-MS) was performed. The constituents of 18 compounds found in CLET with relative peak areas greater than 2% are shown in Table 1. The major components of CLET were n-Hexadecanoic acid (69.7%), Butanoic acid, 3-methyl-, 3,7-dimethyl-6-octenyl ester (6.9%), 1-Octadecyne (5.4%), hexadecanoic acid, and ethyl ester (5.1%) (Figure S3) (Table 1). Furthermore, we found a total 10 compounds occurring in the CLEA fraction. The constituents with relative peak areas are shown in Table 2. The main components of CLEA were dl-2-Phenyltryptophane (29.2%), Benzoic acid, 2,6-dimethyl- (13.7%), 4,7-Methanoazulene, decahydro-1,4,9,9-tetramethyl- (13.3%), and 2-(4-Methyl-2-nitrophenylcarbamoyl)-terephthalic acid (10.2%) (Figure S4) (Table 2).

### 2.4. CLET and CLEA Reduce Intracellular Reactive Oxygen Species (ROS) in High Glucose-Fed C. elegans

**Figure 3:**
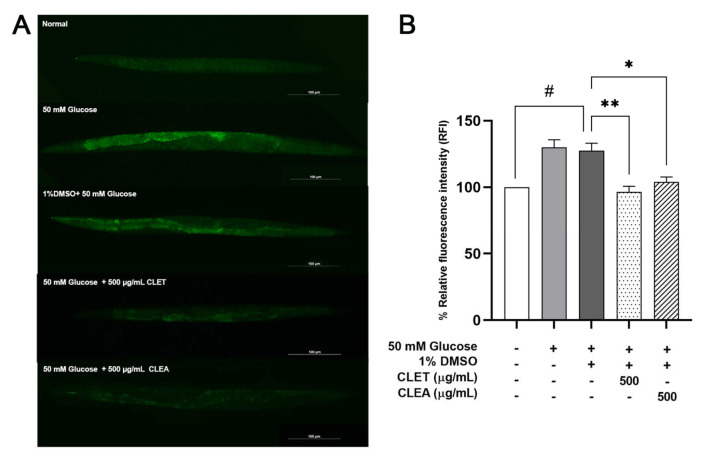
Effect of *C. lentillifera* extracts in reducing the intracellular ROS levels in 50 mM glucose-fed *C. elegans*. (**A**) Fluorescence intensity of H_2_DCFDA of 50 mM glucose-fed worms treated with CL extracts. (**B**) Graphical representation of the relative fluorescence intensity of H_2_DCFDA of 50 mM glucose-fed *C. elegans* treated with CL extracts. The data represent the mean ± SEM (n = 40 per group, number of animals), measured using ImageJ software. Asterisks (*) and (**) indicate significant differences at *p*-values < 0.05 and 0.01. A sharp (#) indicates a significant difference at a *p* value < 0.05 between the glucose-fed and normal groups.

A high concentration of glucose stimulates mitochondrial fission through signals mediated by intracellular Ca^2+^ and ERK1/2, leading to an increase in ROS production [29]. To examine whether CL extracts can reduce the elevated intracellular ROS levels in high-glucose-exposed worms, H_2_DCFDA was used as a fluorescent indicator for measuring cellular H_2_O_2_ levels [30]. We found that high-glucose-fed worms, with or without 1% DMSO, exhibited significant increases in the mean fluorescence intensity compared to the control groups. After the 500 µg/mL CLET and CLEA treatments, significant decreases in the mean fluorescence intensity values were observed (23.91 and 18.26%, respectively) (Figure 3).

### 2.5. CLET and CLEA Did Not Affect the Lifespan of High-Glucose-Fed C. elegans

**Figure 4:**
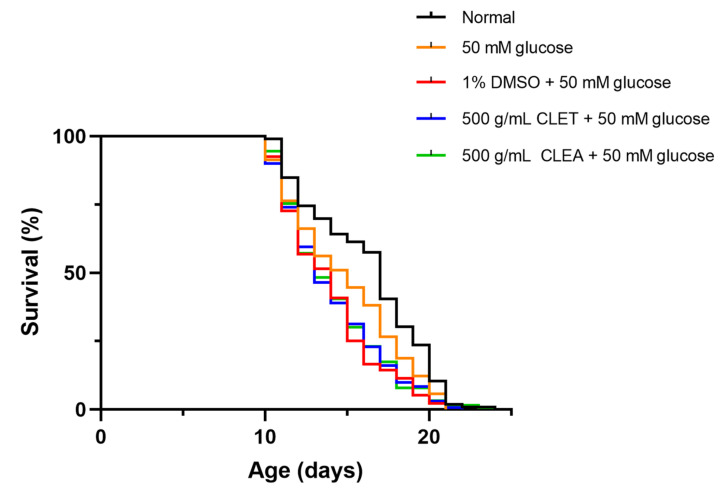
Effect of *C. lentillifera* extracts on the lifespan of 50 mM glucose-fed *C. elegans* compared to the 1% DMSO control.

An adverse effect of high glucose is a shortening of the lifespan. This is caused by mitochondrial dysfunction, leading to increases in ROS production and AGE glycation [31]. The shortened lifespans of high-glucose-fed worms compared to normal worms are shown in Figure 4 and Table 3. The worms treated with 500 µg/mL CLET and CLEA did not show extensions to their lifespans (Figure 4 and Table 3). The mean lifespan of the CL treatment group was not significantly different from that of the glucose-induced group. These results indicate that the extracts of *C. lentillifera* did not affect the lifespan of 50 mM glucose-induced *C. elegans*.

### 2.6. CLET and CLEA Downregulated Levels of mRNA Transcripts of Fatty-Acid-Synthesis-Related Genes

**Figure 5:**
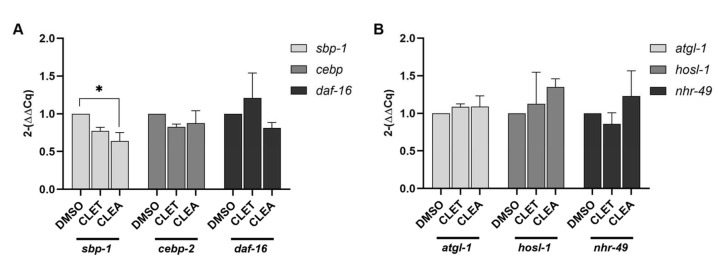
Graphical representations of the comparative fold change of mRNA expression (2^−ΔΔCT^) of fat storage (**A**) and fat metabolism (**B**) genes in 50 mM glucose-fed *C. elegans* treated with *C. lentillifera* extracts. An asterisk (*) indicates a significant difference at a *p*-value < 0.05.

The mRNA expression levels of the lipogenesis genes *sbp-1*, *cebp-2*, and *daf-16* were examined using qRT-PCR. The mRNA expression levels of the *sbp-1* gene of 50 mM glucose-fed worms treated with CLEA were significantly decreased from 1- to 0.64 ± 0.2-fold (*p*-value < 0.05) compared to the 1% DMSO control group, while the expression levels of the *cebp-2* and *daf-16* genes also exhibited slight decreases, without significant differences from those of the control groups (Figure 5A). Moreover, the mRNA expression levels of the lipolysis genes *atgl-1*, *hosl-1*, and *nhr-49* were also determined in the present study. We found that the expression levels of these lipolysis genes were slightly increased, without significant differences from those of the control groups (Figure 5B). Taken together, the CL extracts decreased the total fat accumulation in high-glucose-fed worms by suppressing the *sbp-1* gene.

### 2.7. CLET and CLEA Altered the Expression Levels of GFP-Labeled Lipogenesis and Lipolysis Genes

#### 2.7.1. CLET and CLEA Attenuated GFP-Labeled *sbp-1* Expression Levels

**Figure 6:**
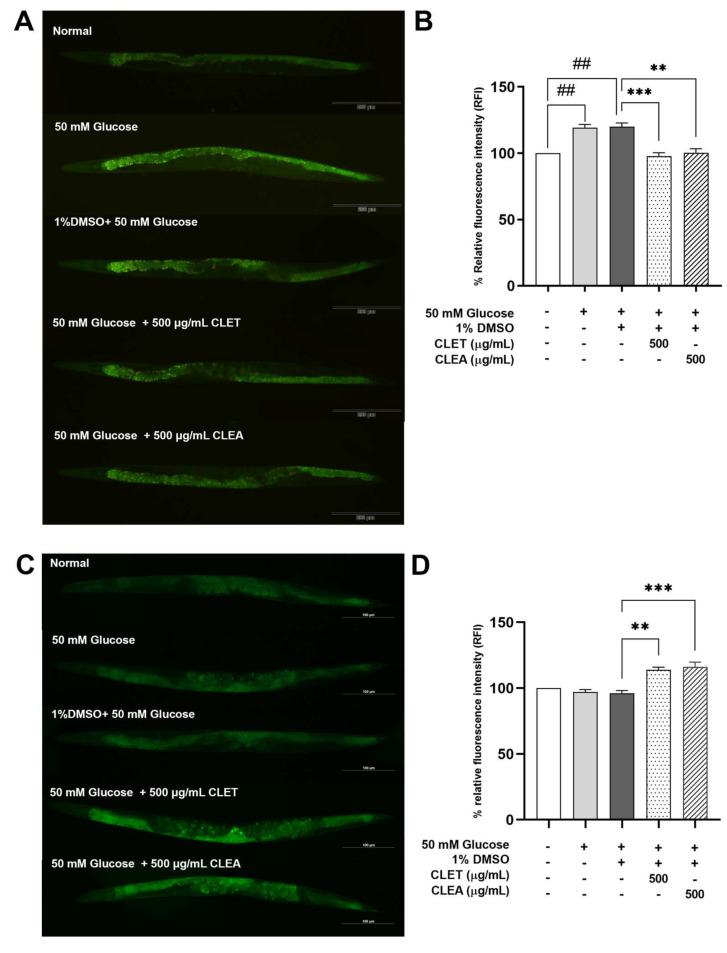
Effect of *C. lentillifera* extracts in terms of decreasing the expression level of the lipogenesis gene *sbp-1* and increasing the level of the lipolysis gene *atgl-1* in 50 mM glucose-fed *C. elegans*. (**A**) Expression pattern of GFP-labeled *sbp-1* gene. (**B**) The relative fluorescence intensity, in percentage, of GFP-labeled *dhs-3* expression. (**C**) Expression pattern of GFP-labeled *atgl-1* gene. (**D**) The relative fluorescence intensity, in percentage, of GFP-labeled *atgl-1* expression. The data represent the mean ± SEM (n = 40 per group, number of animals), measured using ImageJ software. Asterisks (**) and (***) indicate significant differences at *p* values < 0.01 and 0.001. A double sharp (##) indicates a significant difference at a *p*-value < 0.01 between the glucose-fed and normal groups.

We investigated whether CL extracts decreased the expression levels of *sbp-1*, resulting in a reduction in body fat, by analyzing the gene expression in transgenic worms. The *C. elegans* strain CE548 (epEx141[*sbp-1*::GFP::SBP-1 + *rol-6*(su1006)), which carries the GFP-labeled promoter region of *sbp-1,* was used in this experiment. The mean GFP fluorescence intensity values of the GFP-labeled promoter region of *sbp-1* in the CLET and CLEA treatments were significantly decreased to 18.29% and 16.22%, respectively, compared to those of the control groups (Figure 6A,B). This result confirmed that the effect of CL extracts, in terms of lowering fat accumulation, was mediated through the suppression of the *sbp-1* gene.

#### 2.7.2. CLET and CLEA Increased GFP-Labeled *atgl-1* Expression Levels

The VS20 (*atgl-1*p::*atgl-1*::GFP + mec-7::RFP) transgenic worms containing the GFP-labeled promoter region of *atgl-1* were used to measure the gene expression. Figure 6 shows a significant increase in the mean GFP fluorescence intensity of the GFP-labeled promoter region of *atgl-1* in CLET- and CLEA-treated worms of 18.41% and 20.75%, respectively, compared to the control groups (Figure 6C,D), indicating that the CL extracts also activated *atgl-1* expression to reduce the fat accumulation in our *C. elegans* model of obesity.

## 3. Discussion

In the current study, we investigated the effects of extracts derived from *C. lentillifera* on anti-obesity using high-glucose-fed *C. elegans*. Glucose (D-(+)-Glucose) was used to induce fat accumulation in worms in order to establish a model of obesity [25,26,31,32]. High-glucose-fed worms exhibited high levels of total fat accumulation and triglycerides; increased *dhs-3* expression, which is used as a marker of lipid droplets; and shortened the lifespans of the worms. Due to these characteristics, it is believed that *C. elegans* is a suitable model for studying obesity and the anti-obesity effects of extracts from *C. lentillifera*. This study demonstrates that the ethanol and ethyl acetate fractions of *C. lentillifera* (CLET and CLEA, respectively) reduced the total fat deposition, triglyceride content, and intracellular ROS in high-glucose-fed worms. Furthermore, treatments with these extracts reduced the expressions of *sbp-1* mRNA and GFP-labeled *sbp-1,* and increased the expression of GFP-labeled *atgl-1*.

Five fractions of *C. lentillifera* extracts were determined for their fat-accumulation-lowering effects using Nile red dye. The ethanol, ethyl acetate, and aqueous fractions were found to be most effective in reducing fat deposits in high-glucose-fed worms. Consistently, triglyceride levels in glucose-fed worms treated with CL extracts were notably decreased, especially with CLEA. These results indicate that *C. lentillifera* extracts possess bioactive components capable of lowering the total fat and triglyceride levels. This study showed similar results to those reported previously for *Caulerpa racemosa*, the carotenoid extract of which exhibited anti-obesity activity as well as inhibitory effects on α-glucosidase, α-amylase, and antioxidant activity, as determined by DPPH and ABTS assays. Furthermore, the carotenoid extract was able to downregulate TNF-α and mTOR while simultaneously upregulating AMPK [33]. As for *C. lentillifera,* a recent study also demonstrated its anti-obesity potential, particularly through in vitro lipase inhibitory activity. The extract derived from *C. lentillifera* also showed more prominent anti-obesity activity when compared to orlistat, the standard anti-obesity drug [34].

GC-MS analyses revealed that CLEA contains various compounds, including dl-2-phenyltryptophane, which is the most abundant; benzoic acid,2,6-dimethyl-; and butanoic acid,4’-propyl[1,1’-bicyclohexyl]-4-yl ester. The dl-2-Phenyltryptophane is a derivative of tryptophan (Trp) which is a large, neutral amino acid (LNAAs) [35,36]. A disruption in the homeostasis of the Trp/LNAAs ratio in plasma has been proposed as the cause of a phenomenon commonly referred to as carbohydrate-craving syndrome, which is one cause of human obesity [37]. Moreover, Trp is a precursor of the neurotransmitter serotonin (5-HT), which has been found to be associated with obesity. It has been reported that the plasma levels of Trp and 5-HT were low in obese subjects compared to non-obese controls [38,39]. α-Methyl-l-tryptophan (α-MLT) treatment in a mouse model of obesity could diminish the levels of cholesterol and protein in serum, as it has been used as an effective drug for obesity management [40]. Moreover, intragastric administration of Trp in obese men exerts potent effects, suppressing energy intake [41]. Thus, it is of interest to conduct a more in-depth investigation into the anti-obesity potential of dl-2-Phenyltryptophane derived from *C. lentillifera*. Benzoic acid,2,6-dimethyl- is a compound also found in CLEA, and it exhibits functional correlation with a benzoic acid derivative [42]. Benzoic acid (BA) belongs to the category of phenolic compounds, which represents a prominent class of allelochemicals encompassing basic benzoic and cinnamic acid derivatives, quinones, and flavonoids [43]. It is the most frequently employed food preservative, serving as an antimicrobial additive [44,45,46]. BA has been reported to possess the capability to regulate gut functions by enhancing nutrient digestibility in both humans and animals by augmenting the processes of digestion and absorption. This is associated with alterations in mucosal structure, the pH value of the digestive tract, and the synthesis and activity of digestive enzymes in the intestines [45]. Furthermore, 2-Hydroxy 4-methoxy benzoic acid (HMBA) obtained from the roots of *Hemidemus indicus* (*H. indicus*) can reduce the levels of total cholesterol, triglycerides, and LDL-cholesterol in diabetic rats [47]. Furthermore, a previous study reported the antioxidant, anti-diabetic, and anti-obesity properties of four 2-(phenylthio)-ethyl benzoate derivatives (2a). Compound 2a exhibited noteworthy anti-lipase activity, with an IC50 value of 107.95 ± 1.88 µg/mL, as compared to orlistat, which showed an IC50 value of 25.01 ± 0.78 µg/mL [46]. It is possible that the reduction in fat deposition in high glucose-fed worms may have been mediated by the activity of benzoic acid,2,6-dimethyl-. Consequently, further investigation is necessary in order to elucidate the mechanisms of action of this compound.

GC-MS analysis of CLET showed a diverse array of compounds. Among these compounds, two of the most prominent ones were n-hexadecanoic acid (69.76%) and butanoic acid, 3-methyl-, 3,7-dimethyl-6-octenyl ester (6.98%). The n-hexadecenoic acid, commonly referred to as palmitic acid (PA), is a long-chain saturated fatty acid with a straight-chain structure. It is present in various tissues of both animals and humans, as well as in plants, algae, fungi, yeast, and bacteria [48]. Numerous studies have documented the adverse effects of PA, including lipotoxicity. However, most of these studies relied on in vitro cell cultures and animal models of obesity that had been exposed to extremely high concentrations of dietary PA as a single fatty acid, without considering that this condition is not tenable in humans, as dietary PA does not alter its tissue concentration [48]. Based on this conjecture, we believed that the dietary algal extracts containing PA and other similar bioactive compounds may offer beneficial anti-obesity effects. A previous study reported that PA is also present at a high concentration in human milk, as it is synthesized by the activity of the glycerol-3-phosphate (G-3-*p*) acyltransferase, which occurs in the mammary glands. There are a number of studies which have provided compelling evidence demonstrating the protective influence of breastfeeding against the development of obesity during both childhood and adulthood [49,50]. Furthermore, PA also demonstrates a few health benefits, including anti-cancer, anti-Parkinson’s, and antioxidant activities. There was a study reporting that PA inhibits the key molecules of the PI3K/AKT pathway to suppress the proliferation and metastasis of prostate cancer cells [51]. Recently, we found that *Holothuria leucospilota*-derived palmitic acid obtained from an ethyl acetate fraction was able to restore the viability of dopaminergic neurons and decrease α-synuclein aggregation, oxidative stress, and lipid accumulation, as well as extending the lifespan of a *C. elegans* model of Parkinson’s disease [52]. Moreover, the PA obtained from the methanolic extract of *Syzygium litoralle* (Myrtaceae) showed antioxidant activity, with an IC50 value of 44.85 μg/mL [53]. Consistently, PA-enriched *Halymenia durvillei* extract was able to reduce intracellular reactive oxygen species (ROS), as well as to increase the upregulation of mRNA transcripts of antioxidant enzymes in mouse skin fibroblasts (L929) and human immortalized keratinocytes (HaCaT) to promote photoaging activity [54]. Similarly, the present study demonstrates that high-glucose-fed worms treated with CLET, which contains a significant amount of PA, showed decreased intracellular ROS. It is possible that this compound contributed to the amelioration of fat accumulation in high-glucose-fed worms. Butanoic acid, also known as butyric acid, has significant applications in the chemical, food, and pharmaceutical industries [55,56]. Its roles in healthcare are diverse, encompassing a range of bioactive and therapeutic compounds. This includes its utilization in the treatment of hemoglobinopathies; colorectal cancer; gastrointestinal diseases; and metabolic disorders, including obesity [56,57,58]. It is regarded as a safe drug, with therapeutic doses ranging from 150 to 300 mg, and in some cases up to 2000 mg/day, with no demonstrated adverse reactions or clinical side effects [59]. Previously, butanoic acid has been reported to exert anti-obesity properties by improving fasting glycemia, reducing body weight, enhancing insulin tolerance, and improving energy metabolism in animal models [60]. It is in of interest for the future to pursue the purification of these components from CLET and to investigate their structure–activity relationships, particularly in relation to their anti-obesity properties. Taken together, the extracts from *C. lentillifera* contain numerous active compounds that contribute to anti-obesity effects, effectively reducing fat accumulation in high-glucose-fed *C. elegans*, which serves as a model of obesity.

Although the intracellular ROS levels were significantly decreased, the lifespans of worms treated with 50 mM glucose were not restored by treatments with CL extracts. Consistently, as estimated by qRT-PCR, the mRNA expression levels of *daf-16* were not significantly different from the 1% DMSO control group. It is indicated that CL extracts were unable to increase the activation of *daf-16* to promote longevity in high-glucose-fed worms. On the other hand, we found that the extracts from *C. lentillifera* extended the lifespans of *C. elegans* in normal conditions (Figure S2 and Table S1). It is possible that a high concentration of glucose may suppress the lifespan-extending actions of the compounds contained in CL extracts. However, the precise mechanisms behind this phenomenon should be further examined. An ortholog of the mammalian SREBP (sterol regulatory element-binding protein), the *sbp-1* gene, plays a crucial role in the synthesis of fatty acids, triglycerides, and cholesterol. It is a transcription factor regulating the expression of the genes encoding lipogenic enzymes in both worms and mammals [26]. In qRT-PCR, the mRNA expression of the *sbp-1* gene was decreased in high-glucose-fed worms treated with CL extracts. This result was confirmed by a GFP-labeled *sbp-1* expression assay, which showed a significant decrease in GFP intensity in the high-glucose-fed *C. elegans* that were treated with CL extracts. Likewise, a previous study also reported that the ethanolic extract of *Caulerpa okamurae* inhibited lipid accumulation by reducing the expression of the master regulator of adipogenesis, PPARγ, SREBP1c, and C/EBPα in 3T3-L1 adipocytes [61]. It is plausible that the compounds present in *C. lentillifera* extracts might negatively modulate the *sbp-1* gene, leading to a reduction in fat accumulation in high-glucose-fed *C. elegans*. Mammalian CCAAT/enhancer-binding proteins (C/EBPs) act as regulators in adipocyte differentiation. The *cebp-2* gene, a homolog of C/EBPs, governs fat deposition and the desaturation of fatty acid in *C. elegans* [62]. Unexpectedly, there was no significant difference in *cebp-2* expression with the CL treatment compared to 1% DMSO control worms, suggesting that CL extracts do not mediate *cebp-2* when lowering fat accumulation. However, it was observed that the expression levels of lipolysis genes (*atgl-1*, *hosl-1*, and *nhr-49*) remained unchanged. These results suggest that the decrease in fat accumulation induced by CL extracts in high-glucose-fed worms occurred independently of lipolysis gene activity. With the results taken together, our study demonstrates that the extracts, particularly CLET and CLEA, were able to exert anti-obesity activities through the deactivation of the *sbp-1* gene. In the near future, we aim to evaluate the activities of purified compounds from CL extracts to further understand their mechanisms of action. Meanwhile, our present study offers strong evidence suggesting that CL extracts, particularly CLET and CLEA, may be used as novel therapeutic agents or food supplements for combating obesity.

## 4. Materials and Methods

### 4.1. C. elegans Strains and Maintenance

*C. elegans* worms were provided by *Caenorhabditis* Genetics Center (CGC) at the University of Minnesota, USA. Bristol N2 (wild-type), CE548 (epEx141[*sbp-1*::GFP::SBP-1 + *rol-6*(su1006)), VS20 (*atgl-1*p::*atgl-1*::GFP + *mec-7*::RFP), and LIU-1 (*dhs-3p*::*dhs-3*::GFP + *unc-76*(+)) strains of *C. elegans* were used as model organisms in this study. The worms were cultured using the standard method [63] on solid nematode growth medium (NGM) agar plates, containing a lawn of living *Escherichia coli* bacteria, strain OP50, as a food source at 20 °C.

### 4.2. Establishment of Obesity Model in C. elegans

In this study, the induction of excess fat accumulation in wild-type N2 *C. elegans* was performed by feeding them with high levels of glucose. The glucose solutions were obtained by dissolving D(+)-glucose (AppliChem, Darmstadt, Germany) with OP50 bacteria. Glucose, at a concentration of 50 mM, was shown to be the most appropriate concentration inducing fat accumulation to establish an obese *C. elegans* model [25].

### 4.3. The Extraction and Analysis of Caulerpa lentillifera

The macroalgae *C. lentillifera* obtained from the Phetchaburi Coastal Aquaculture Research and Development Center, Phetchaburi, Thailand, were collected and dried before extraction. The authentication of *C. lentillifera* was conducted by Dr. Montakan Tamtin at the Coastal Aquatic Feed Research Institute and Coastal Fisheries Research and Development Bureau, Petchaburi, Department of Fisheries, Thailand. The dried samples of *C. lentillifera* were mashed and extracted by maceration. The samples were manually pressed while marinating with 95% ethanol at room temperature for 7 days, and were subsequently evaporated. Afterward, an ethanol fraction was continually partition- extracted using hexane, ethyl acetate, butanol, and distilled water, respectively. All fractions were obtained and dissolved in DMSO diluted with the medium to obtain the proper concentrations used for our *C. elegans* model of obesity.

### 4.4. Gas Chromatography–Mass Spectrometry (GC-MS) Analysis

The bioactive compounds contained in the ethanol and the ethyl acetate fractions of *C. lentillifera* extracts were identified using GC-MS analysis at the chemistry department of King Mongkut’s University of Technology Thonburi, Bangkok, Thailand. Briefly, these two fractions were analyzed using an AGILENT GC-7890B/MSD-5977A system with a standard non-polar column HP-5 size of 30 mm × 0.25 mm ID × 0.25 µm film thickness. The compounds were further identified by blasting with the National Institute of Standards and Technology (NIST) Mass Spectrometry Data Center.

### 4.5. Dose–Response Assessment

The concentrations of the extracts derived from *C. lentillifera* were investigated via a dose–response assessment. Synchronized L1 *C. elegans* larva were transferred into NGM plates containing the lawn OP50, then incubated at 20 °C for 48 h. After 48 h, the L4 stage worms were collected and soaked in S-medium with the extracts at doses of 0, 100, 250, 500, 750, and 1000 µg/mL for 1 h. Afterward, the worms were centrifuged at 1500 RPM for 3 min and washed with M9 buffer 2–3 times. They were transferred into NGM plates containing OP50 and incubated at 20 °C for 24 h. Surviving and dead worms were counted. The lethal dose at 50% (LD50) was analyzed and presented in a dose–response curve.

### 4.6. Nile Red Staining Assay

The effect of *C. lentillifera* extracts on body fat accumulation was measured by the 9-diethylamino-5H-benzo[α]phenoxazine-5-one, Nile red (Sigma-Aldrich, St. Louis, MO, USA), which is a fluorescent dye stain for lipid droplets, in the intestinal cells of *C. elegans* [64]. The synchronized L1 larvae were divided into groups: a normal group (only fed with OP50 bacteria), an induced group (treated with 50 mM glucose without DMSO), a control group (treated with 50 mM glucose and 1% DMSO), and the extract-treated groups. The synchronized L1 larvae were washed with M9 buffer and centrifuged at 1500 rpm for 3 min to remove the supernatant. About 50 worms were transferred into the NGM plate containing the lawn OP50 bacteria, which was mixed with 50 mM glucose and Nile red fluorescence dye at a ratio of 250:1. After they reached the L4 larvae stage, worms were transferred into a NGM/FUDR plate containing the same contents as the NGM plates. FUDR is an inhibitor of DNA synthesis, and was able to prevent the hatching of eggs in the experimental plates [65]. L4 stage worms were cultured at 20 °C for 2 days. Afterward, the adult worms were collected and transferred onto slides overlaid with 2% agar pads. The fluorescence intensity was observed under a fluorescence microscope (BX-53; Olympus Crop., Tokyo, Japan).

### 4.7. Triglyceride Quantification Assay

A triglyceride quantification kit (Biovision, Waltham, MA, USA) was used to measure the triglyceride (TG) contents. The synchronized L1 larvae were fed with 50 mM glucose and 500 µg/mL CLET, CLEA, and CLAQ extracts on NGM plates for 2 days. Worms growing into L4 larvae were transferred into NGM/FUDR plates. After that, the adult worms were collected by washing with M9 buffer, then centrifuged to remove the supernatant. In the triglyceride assessment, worms from each condition was added to 1 mL of 5% NP-40 in water and homogenized on ice. Then, the samples were slowly heated twice to solubilize all triglycerides and centrifuged at maximum speed for 2 min to remove insoluble contents. The soluble samples were used for the measurement of triglyceride contents, following the manufacturer’s instructions. The experimental 96-well plate was incubated for 60 min in the dark at room temperature. Afterward, a colorimetric assay was performed to measure the absorbance at 570 nm using the microplate reader.

### 4.8. Intracellular ROS Levels Analysis

After exposure to 50 mM glucose and treatment with 500 µg/mL of CLET and CLEA for 96 h, the intracellular ROS levels of the worms were analyzed using H_2_DCF-DA. The worms were incubated with 25 µM H_2_DCF-DA in the dark for 60 min at room temperature. The ROS production of each group of worms was investigated by imaging under a fluorescence microscope (ECLIPSE Ci-L, Nikon Corp., Tokyo, Japan). The ROS levels were quantified in a minimum of 30 worms. The fluorescence intensity was analyzed using ImageJ software. The experiment was independently repeated three times.

### 4.9. Lifespan Assay

After synchronization, the wild-type L1 worms were transferred into NGM plates containing OP50 mixed with 50 mM glucose and the *C. lentillifera* extracts (treated group). One group was treated with only 50 mM glucose (control group without DMSO), one was treated with 50 mM glucose and 1% DMSO (control group), and one was only fed with OP50 bacteria (normal group). Until the worms reached the L4 stage, they were transferred into NGM/FUDR plates with the same contents as the NGM plates. The worms were incubated at 22 °C and counted daily as alive, dead, and censored until all of the worms had died. The results were analyzed statistically using the Prism-GraphPad program.

### 4.10. Quantitative RT-PCR

The adult worms were collected in order to extract the total RNA of the worms using an RNAeasy Qiagen kit (Qiagen, Hilden, Germany). The concentrations of RNA were measured by Nanodrop™ (Thermo Fisher Scientific, Waltham, MA, USA) and kept at −80 °C. The RNA samples were converted to generate cDNA by iScript™ Reverse transcription (Bio-Rad, Hercules, CA, USA), following the manufacturer’s protocol. SYBR green real-time PCR analyses were performed using SsoFast™ EvaGreen^®®^ Supermix with Low ROX qRT-PCR (Bio-Rad, Hercules, CA, USA). The forward and reverse primers are shown in Table 4. The optimized cycling conditions for qPCR were started at enzyme activation at 95 °C for 30 s (1 cycle), followed by 44 cycles of reaction sets at 95 °C for 5 s, 60 °C for 30 s, and finally at 95 °C to end the reaction. Evagreen fluorescence was detected using a real-time PCR detection system (Bio-Rad, Hercules, CA, USA). The Cq value of each gene was calculated with the comparative 2^(−ΔΔCq)^ method, and the data were presented as fold changes. Actin (*act-1*) was used as the endogenous control. The assay was repeated in triplicate using independent RNA preparation.

### 4.11. Quantification of GFP-Labeled Gene Expression Levels

The strains used in this assay were CE548 (*epEx141[sbp-1::GFP::SBP-1 + rol-6(su1006)]*), VS20 (*atgl-1p::atgl-1::GFP + mec-7::RFP*), and LIU-1 (*dhs-3p::dhs-3::GFP + unc-76(+)*), which carry green fluorescence protein (GFP) tags at the promotor regions of *sbp-1*, *atgl-1,* and *dhs-3*, respectively. The synchronized L1 larvae were treated with 50 mM glucose and *C. lentillifera* extracts mixed with the lawn OP50 bacteria, then incubated at 20 °C for 4 days. After that, adult worms were collected, and the fluorescence intensities of *sbp-1*::GFP, *atgl-1*::GFP, and *dhs-3*::GFP were quantified under a fluorescence microscope (ECLIPSE Ci-L, Nikon Corp., Tokyo, Japan).

### 4.12. Statistical Analysis

The statistical analysis was performed using GraphPad Prism software (GraphPad Software, Inc., San Diego, CA, USA). All of the results of the treated groups and their control groups were presented as the mean ± SEM and analyzed by one way-ANOVA, as well as the Turkey–Karma test for multiple comparisons. The lifespans were analyzed using Kaplan–Meier survival curves and log-rank tests. Statistical significance was set at *p* values ˂ 0.05, 0.01, 0.001.

## 5. Conclusions

The present study has demonstrated that ethanol and ethyl acetate extracts derived from *C. lentillifera* contain various bioactive compounds capable of reducing the accumulation of fat, triglycerides, and *dhs-3-*positive lipid droplets in high-glucose-fed worms. In addition, CL extracts also decreased intracellular ROS levels. These effects were demonstrated to be mediated through the *sbp-1* pathway. Hence, CLET and CLEA exhibit potential as pharmaceutical components or food supplements for combating obesity. However, the precise mechanisms underlying these effects necessitate further investigation in order to develop CLET, CLEA, and purified compounds derived from these fractions as potential forms of nutritional therapy and drugs for the treatment of obesity.

## Figures and Tables

**Table 1:** Major compounds analyzed in CLET via GC-MS analysis.

	Compounds	RT (min)	MW	Molecular Formula	Peak Area	% Peak Area
1	n-hexadecanoic acid	33.51	256.24	C_16_H_32_O_2_	56911251	69.76
2	Butanoic acid, 3-methyl-, 3,7-dimethyl-6-octenyl ester	36.312	240.209	C_15_H_28_O_2_	5697899	6.98
3	Hexadecanoic acid, ethyl ester	34.084	284.272	C_18_H_36_O	4160343	5.10
4	1-Octadecyne	31.895	250.266	C_18_H_34_	3001459	3.68
5	.alpha.,.alpha.,.alpha.’,.alpha.’-Tetramethyl-1,4-benzenedimethanol	5.526	194.131	C_12_H_18_O_2_	1984896	2.43
6	Hexadecanal	30.576	240.245	C_16_H_32_O	1707579	2.09
7	Citronellyl isobutyrate	31.537	226.193	C_14_H_26_O_2_	1690647	2.07

RT: retention time; MW: molecular weight.

**Table 2:** Major compounds analyzed in CLEA via GC-MS analysis.

	Compounds	RT (min)	MW	Molecular Formula	Peak Area	% Peak Area
1	dl-2-Phenyltryptophane	43.544	280.121	C_17_H_16_N_2_O_2_	1655848	29.28
2	Benzoic acid, 2,6-dimethyl-	6.562	150.068	C_9_H_10_O_2_	779649	13.79
3	4,7-Methanoazulene, decahydro-1,4,9,9-tetramethyl-	31.028	206.203	C_15_H_26_	752012	13.30
4	2-(4-Methyl-2-nitrophenylcarbamoyl)-terephthalic acid	33.491	344.064	C_16_H_12_N_2_O_7_	579464	10.25
5	4-Chloro-6-(2-hydroxyphenyl)pyrimidine	11.949	206.025	C_10_H_7_ClN_2_O	405552	7.17
6	Tetradecahydro-1-methylphenanthrene	31.895	206.203	C_15_H_26_	388071	6.86
7	1-(4-Methoxyphenyl)imidazoline-2-thione	46.774	206.051	C_10_H_10_N_2_OS	314169	5.56
8	Pyrimidin-2-amine, 4-(2,4-dimethyl-5-thiazolyl)-	4.429	206.063	C_9_H_10_N_4_S	314395	5.56
9	(S)-3-Ethyl-4-methylpentanol	3.878	130.136	C_8_H_18_O	294186	5.20
10	Butanoic acid, 4’-propyl[1,1’-bicyclohexyl]-4-yl ester	43.261	294.256	C_19_H_34_O_2_	171253	3.03

RT: retention time; MW: molecular weight.

**Table 3:** Mean lifespan; number of worms; and percentage by which the lifespans of wild-type N2, 50 mM glucose-fed worms increased when treated with 1% DMSO or *C. lentillifera* extracts.

Strain	Mean Lifespan (Days)	No. of Worms	Increase in Lifespan (%)	*p*-Value
N2	16.2 ± 3.557	106	-	
N2 + 50 mM glucose	14.09 ± 2.932	122	−13.02	<0.0001
N2 + 50 mM glucose + 1% DMSO	13.89 ± 2.938	132	-	
N2 + 50 mM glucose + 500 µg/mL CLET	14.02 ± 3.121	131	0.94	ns
N2 + 50 mM glucose + 500 µg/mL CLEA	14.08 ± 3.090	126	1.37	ns

**Table 4:** Forward and reverse primers used for qRT-PCR analysis.

Genes	Forward Primer (5′ To 3′)	Reverse Primer (5′ To 3′)
*sbp-1*	TCCATACGACCAAGCTCAAGG	TGCCACTTGTTCAGGGTTCT
*cebp-2*	TCGGAAGCGAAACACATCAGA	TTCTGTAGCTGCTCGACCTTT
*daf-16*	TTCCTGAAGAAGATGCTGACCTA	TATCGTCTGGCGATTCGGAC
*atgl-1*	TTATCCGCAGTTTCCGCTCC	ACATGCATCACGTTTACTTGTAGT
*hosl-1*	CAACTTCAGGACCACTGGGG	GTTGTCCGAACACATCTGTACTA
*nhr-49*	CCATGCACAACCGAGGATCT	TCGAGTTGTATCGGGACTTCG
*act-1*	CAATCTACGAAGGATATG	ATGAGGTAATCAGTAAGA

## Data Availability

The data supporting the conclusions of this study are available upon request from the corresponding author.

## Supplementary Materials

The following supporting information can be downloaded at: https://www.mdpi.com/article/10.3390/md21110577/s1, Figure S1: Toxicity of five fractions derived from *C. lentillifera*; Figure S2: Effect of *C. lentillifera* extracts on the lifespan of *C. elegans*; Figure S3: GC-MS Chromatogram of CLET; Figure S4: GC-MS Chromatogram of CLEA; Table S1. Mean lifespan, number of worms, and percentage of increase lifespan of wild type N2, 50 mM glucose-fed worms treated with 1% DMSO or *C. lentillifera* extracts.
