# Supplementary material for: Anti-Obesity Effects of Marine Macroalgae Extract Caulerpa lentillifera in a Caenorhabditis elegans Model
Source: Mar Drugs. 2023 Nov 3;21(11):577. doi: 10.3390/md21110577 (PMC10672060; doi:10.3390/md21110577)
Supplement: Supplementary file 1 [file marinedrugs-21-00577-s001.zip › marinedrugs-2663324-supplementary.pdf]

Supplementary data for Manuscript entitle

"Anti-obesity Effects of the Marine Macroalgae Extracts, *Caulerpa lentillifera*, in *Caenorhabditis elegans* Model"

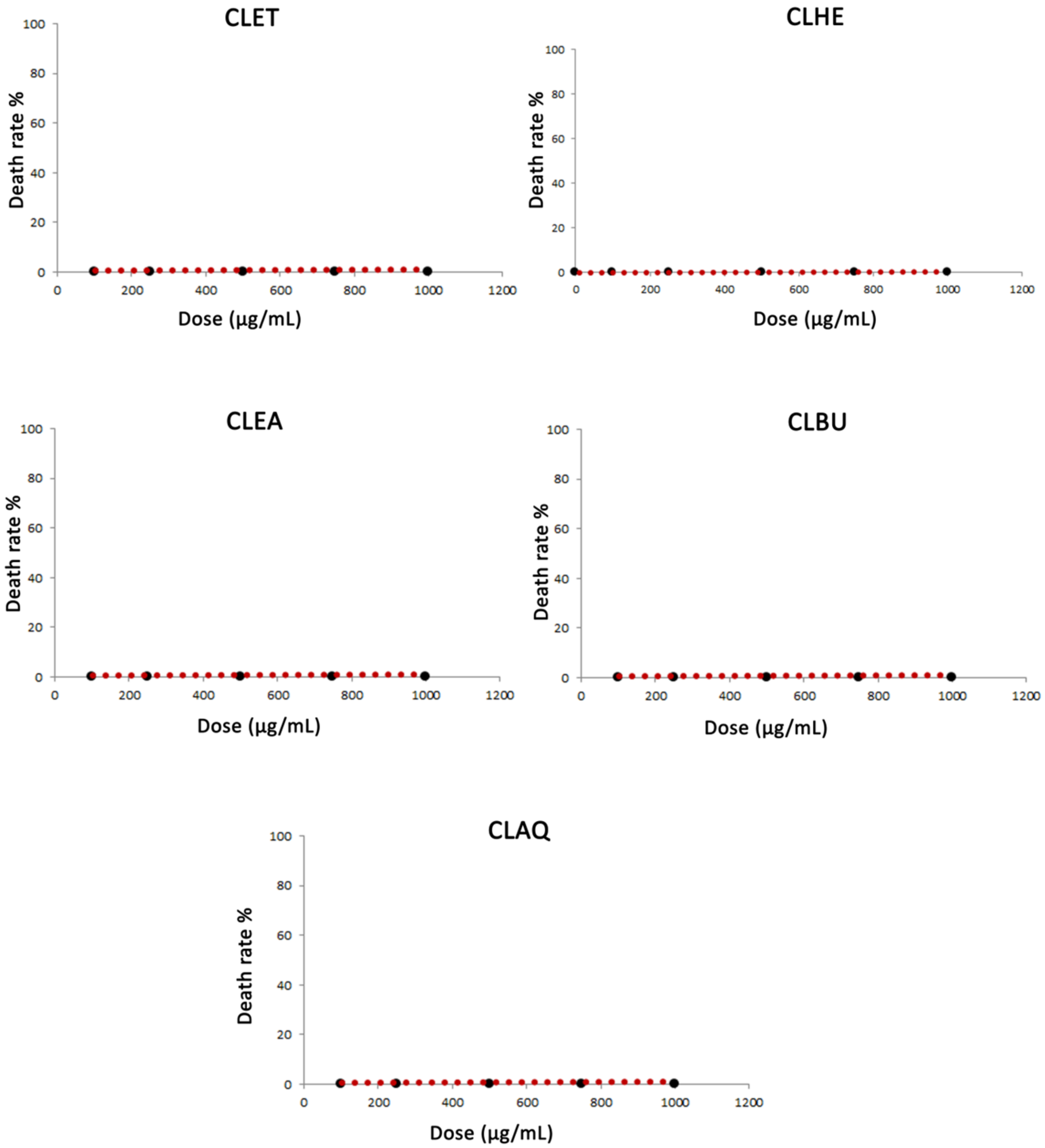

Figure S1. Toxicity of five fractions derived from *C. lentillifera* including ethanol (CLET), hexane (CLHE), ethyl acetate (CLEA), butanol (CLBU), and aqueous (CLAQ). The CL extracts at concentration of 100, 250, 500, 750, and 1000 µg/mL, showed 100% survival rates. This indicates that all fractions of CL extracts are non-toxic to *C. elegans*.

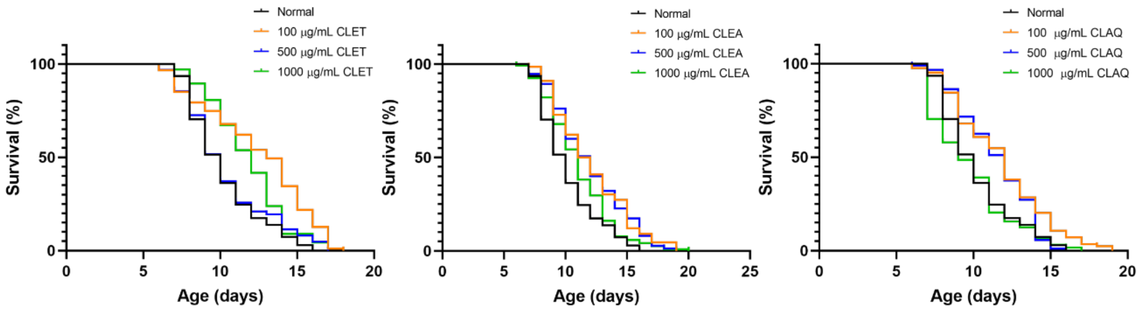

Figure S2. Effect of *C. lentillifera* extracts on the lifespan of *C. elegans* compared to the normal worms group.

Table S1. Mean lifespan, number of worms, and percentage of increase lifespan of wild type N2, 50 mM glucose-fed worms treated with 1% DMSO or *C. lentillifera* extracts.

Strain	Mean lifespan (days)	No. of worms	Increase in lifespan (%)	P value
N2	10.25 ± 2.54	115		
N2 + 100 μg/mL CLET	12.39 ± 3.51	87	20.88	<0.0001
N2 + 500 μg/mL CLET	10.34 ± 2.89	62	0.88	0.9933
N2 + 1000 μg/mL CLET	11.76 ± 2.46	67	14.73	0.0029
N2 + 100 μg/mL CLEA	12.03 ± 3.06	66	17.37	0.0002
N2 + 500 μg/mL CLEA	11.95 ± 3.02	75	16.59	0.0003
N2 + 1000 μg/mL CLEA	11.02 ± 2.68	118	7.51	0.2012
N2 + 100 μg/mL CLAQ	11.71 ± 3.06	84	14.24	0.0016
N2 + 500 μg/mL CLAQ	11.39 ± 2.38	88	11.12	0.0259
N2 + 1000 μg/mL CLAQ	9.75 ± 2.66	64	-4.88	0.8699

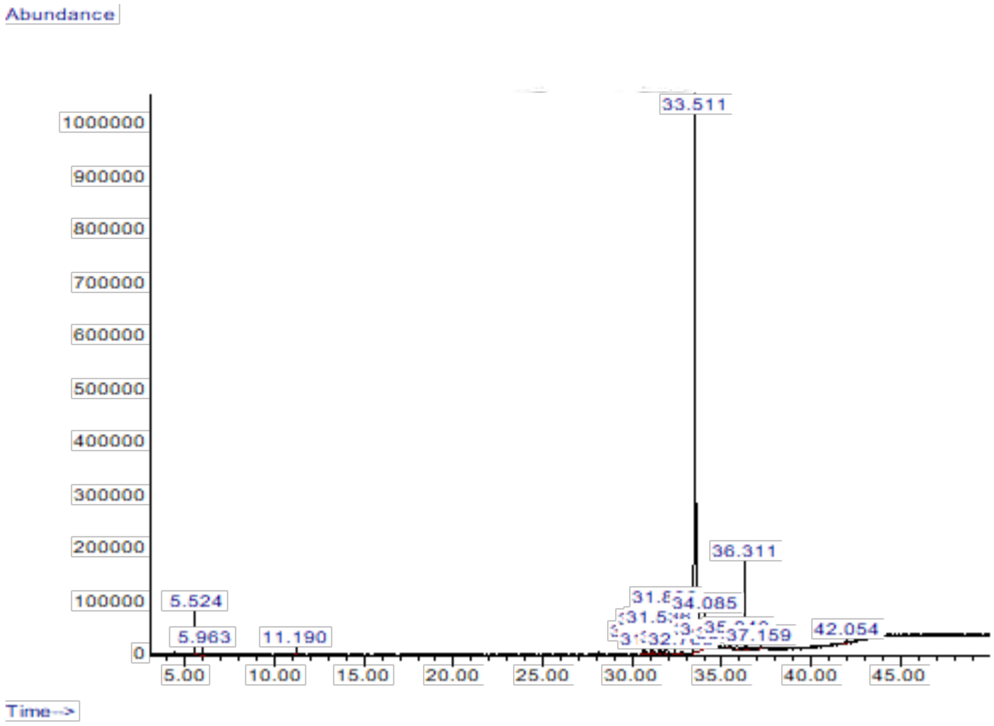

Figure S3. GC-MS Chromatogram of CLET. Three of the most abundant compounds include n-Hexadecanoic acid (69.76%), Butanoic acid, 3-methyl-, 3,7-dimethyl-6-octenyl ester (6.98%), and Hexadecanoic acid, ethyl ester (5.10%).

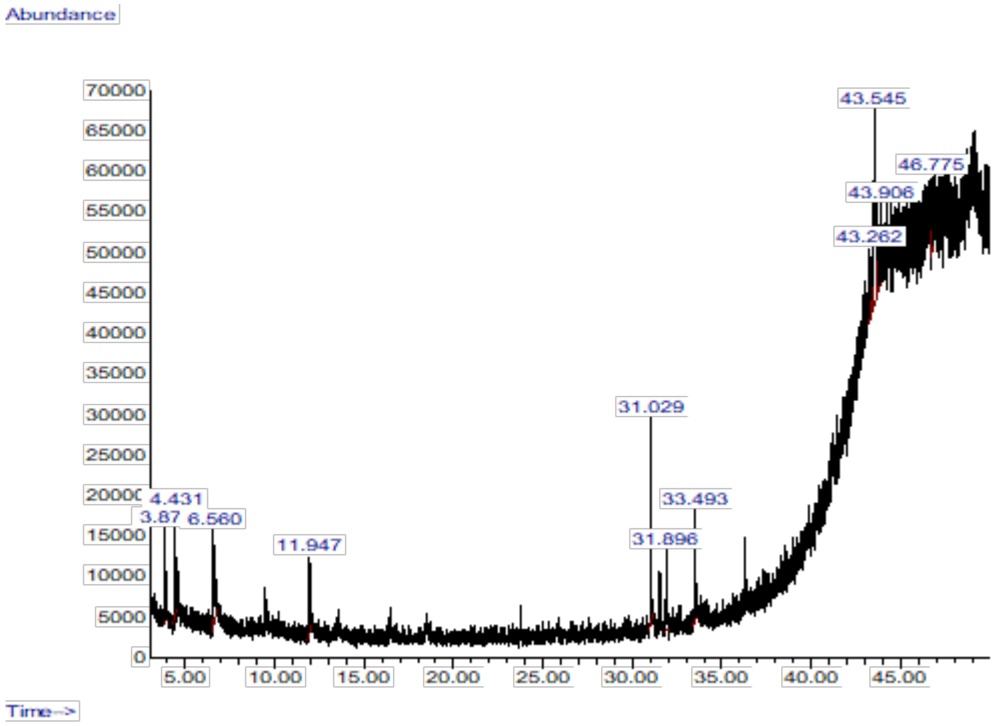

Figure S4. GC-MS Chromatogram of CLEA. Three of the most abundant compounds include dl-2-Phenyltryptophane (29.28%), Benzoic acid, 2,6-dimethyl- (13.79%), and 4,7-Methanoazulene, decahydro-1,4,9,9-tetramethyl- (13.30%).
